# The specific applications of the TSR-based method in identifying Zn^2+^ binding sites of proteases and ACE/ACE2

**DOI:** 10.1016/j.dib.2022.108629

**Published:** 2022-09-23

**Authors:** Titli Sarkar, Camille R. Reaux, Jianxiong Li, Vijay V. Raghavan, Wu Xu

**Affiliations:** a The Center for Advanced Computer Studies, University of Louisiana at Lafayette, Lafayette, LA 70504, USA; b Department of Chemistry, University of Louisiana at Lafayette, PO Box 44370, Lafayette, LA 70504, USA; c High Performance Computing, Frey Computing Services Center, Louisiana State University, Baton Rouge, LA 70803, USA

**Keywords:** TSR, Metal ion binding site, Protein similarity, 3D structure, Alignment-free, Structural motif, Structure comparison, Amino acid grouping

## Abstract

We have developed an alignment-free TSR (Triangular Spatial Relationship)-based computational method for protein structural comparison and motif identification and discovery. To demonstrate the potential applications of the method, we have generated two datasets. One dataset contains five classes: Actin/Hsp70, serine protease (chymotrypsin/trypsin/elastase), ArsC/Prdx2, PKA/PKB/PKC, and AChE/BChE at the hierarchical level 1 and twelve groups at the level 2. The other dataset includes representative proteases and ACE/ACE2. The x,y, z coordinates of the structures were obtained from PDB. We calculated the keys (or features) that represent each structure using the TSR-based method. The dataset and data presented here include additional information that help the readers become aware of specific applications of the TSR-based method in protein clustering, identification and discovery of metal ion binding sites as well as to understand the effect of amino acid grouping on protein 3D structural relationships at both global and local levels.

**Specifications Table**

**Table:** 

Subject	Biological Sciences: Bioinformatics and Computational Biology
Specific subject area	Development of protein 3-D structural comparison methods for BLAST and understanding of relations between protein sequences, structures and functions
Type of data	List the type(s) of data this article describes. Table Image Chart Graph Figure Triplet and key files: Generated using the TSR-based algorithm
How data were acquired	The 3-D structure data are from PDB (https://www.rcsb.org/). We calculated the keys for each structure using the TSR-based computational method that is available from GitHub.
Data format	List your data format(s) Raw Analyzed Filtered
Parameters for data collection	MaxDist, Theta, and Three Amnio Acids and Their Positions
Description of data collection	For every protein, C_α_ atoms from its PDB file were selected. All three edge lengths and angles of all possible triangles formed by C_α_ were calculated. The labels, lengths and angles were formulated to keys using the TSR-based algorithm.
Data source location	Institution: University of Louisiana at Lafayette City/Town/Region: Lafayette, Louisiana Country: USA Latitude and longitude (and GPS coordinates, if possible) for collected samples/data: 30.2238889 and -92.0197222 Primary data sources: Protein Data Bank
Data accessibility	With the article
Related research article	Titli Sarkar^2^, Vijay V. Raghavan^2^, Feng Chen^3^, Andrew Riley^2^, Sophia Zhou^1,†^ and Wu Xu^1,*^, Exploring the effectiveness of the TSR-based protein 3-D structural comparison method for protein clustering, and structural motif identification and discovery of protein kinases, hydrolases, and SARS-CoV-2’s protein via the application of amino acid grouping, *Computational Biology and Chemistry*. https://www.sciencedirect.com/science/article/abs/pii/S1476927121000463

## Value of the Data

• Common set of local structures among proteins, especially from structurally unrelated proteins, often provides some of the most striking consequences regarding protein functions. To increase structural diversity, we generated a dataset with five classes: Actin/Hsp70, serine protease (chymotrypsin/trypsin/elastase), ArsC/Prdx2, PKA/PKB/PKC, and AChE/BChE at the hierarchical level 1 and twelve groups at the level 2.
• The data will help the readers, who are interested in the relation of protein 3D structure and function and who are in the field of computational biology or chemistry or bioinformatics, to understand the potential applications of the TSR-based method in protein clustering, and identification and discovery of metal ion binding sites.
• We have provided the details of the data (PDB IDs, key generation formula and algorithms we have used). The dataset, data, and source code will help the researchers in the field to use the TSR-based method in their own research.

## 1 Data Description

### 1.1 The dataset contains five protein classes with high diversity of sequence and structure to enable the study of the effect of amino acid grouping on hierarchical clustering when using the TSR-based method

**Fig. 1:**
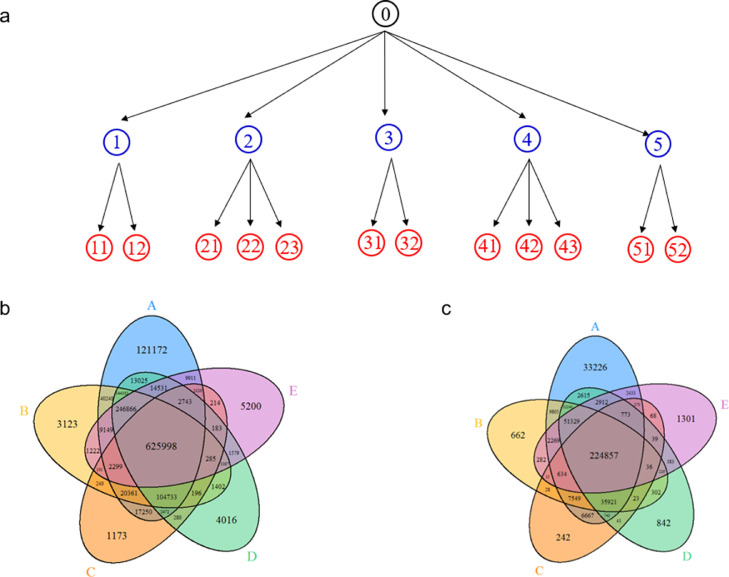
The hierarchical organization of the structure dataset and Venn diagram analysis of the structures at the hierarchical level 1 for with and without amino acid grouping. a, The hierarchical organization of the structure dataset with five classes at the level 1 and twelve subclasses at the level 2 (leaf node level); b**-c**, The Venn diagram shows counts of the keys **without (b) and with (c) amino acid grouping** that are specific to each class of the structures at the level 1, and all possibly overlapped regions**.** The numbers of Common and the total keys and their ratios, for without **(b)** and with **(c)** amino acid grouping, are indicated.

**Fig. 2:**
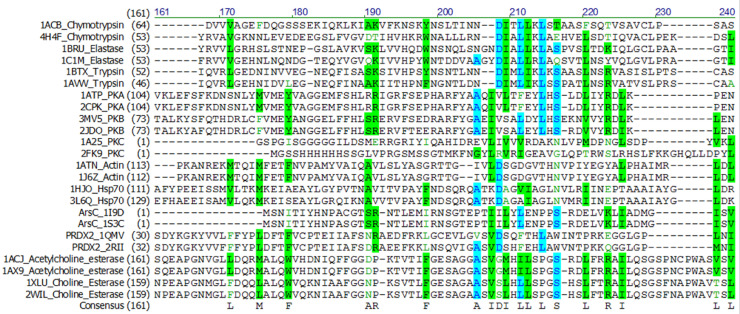
The sequence alignment of the representative proteins from chymotrypsin, elastase, trypsin, PKA, PKB, PKC, Actin, Hsp70, Arsc, PRDX2, acetylcholine esterase, and choline esterase.

**Fig. 3:**
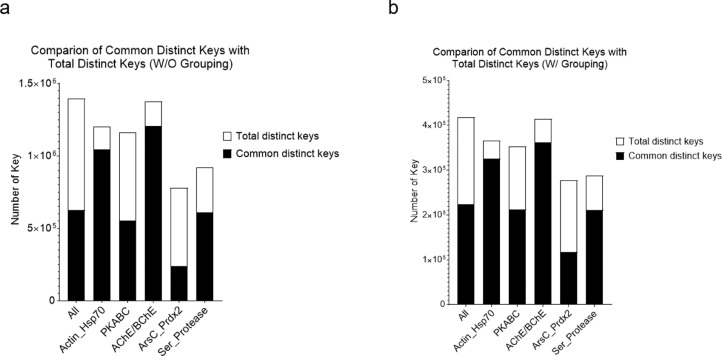
Comparison of the number of the Common distinct keys with the number of the total distinct keys of the structures at the hierarchical level 1. a, Without amino acid grouping; b, With amino acid grouping.

**Fig. 4:**
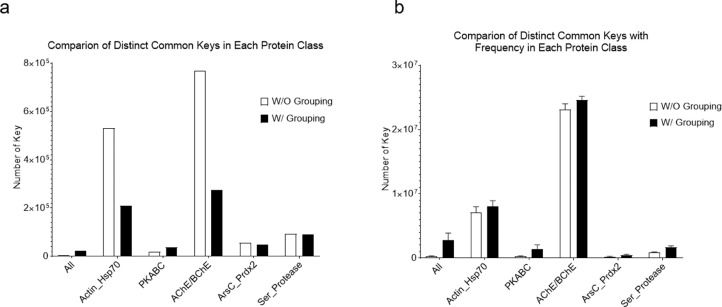
Effect of amino acid grouping on common keys in each of five different protein families. a, The numbers of the common distinct keys without consideration of key frequency were calculated and are present for with and without amino acid grouping; b, The numbers of the common distinct keys with consideration of key frequency were calculated and are present for with and without amino acid grouping.

**Fig. 5:**
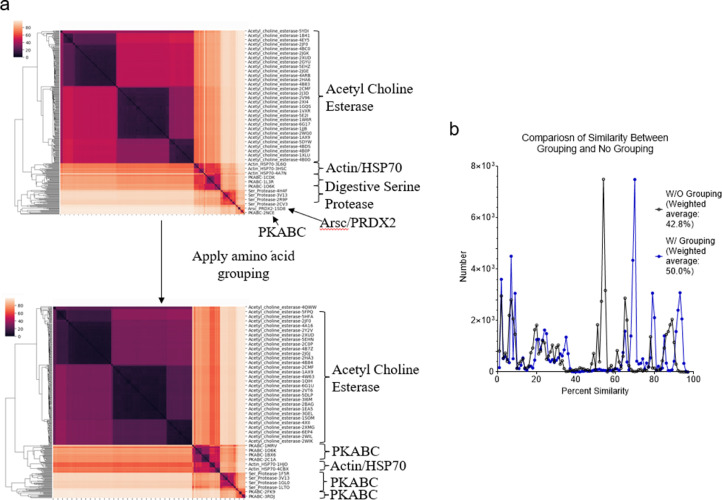
Clustering and structure similarity of the dataset without amino acid grouping compared with amino acid grouping. a, Clustering comparison between without and with amino acid grouping; b, Comparison of similarity distributions between with and without amino acid grouping. The weighted averages are indicated.

We have developed the TSR (Triangular Spatial Relationship)-based method for protein structural comparison [1], [2], [3]. In this method, all possible triangles are constructed with C_α_ atoms of a protein as vertices. Every triangle is represented by an integer denoted as a "key" computed through the TSR key computation algorithm. The 3D structure of each protein is thereby represented by a vector of integers. Identification of common local structures among proteins, especially from structurally unrelated proteins, often provides some of the most striking consequences regarding protein functions. To increase structural diversity while studying the effect of amino acid grouping on hierarchical clustering, we generated a dataset with five classes: Actin/Hsp70, serine protease (chymotrypsin/trypsin/elastase), ArsC/Prdx2, PKA/PKB/PKC, and AChE/BChE at the hierarchical level 1 and twelve groups at the level 2 (Fig. 1a). The Venn diagram shows that five classes/twelve groups share 44.8% (625,998 distinct Common keys out of 1,396,530 total distinct keys) of the distinct Common keys (Fig. 1b). Amino acid grouping decreases the total numbers of distinct keys and distinct Common keys, while it increases the percent of distinct Common keys (224,857/418,743 = 53.7%) (Fig. 1c). A representative sequence alignment of these five classes of proteins shows little amino acid sequence similarity, implying substantial structure diversity of the dataset (Fig. 2). We have learned that five classes have a high percent of distinct Common keys. To understand the Common keys at the hierarchical level 1, we calculated the percentages for the five individual classes. The order of the Common keys from high to low is: AChE/BChE (1,206,051/1,376,159 = 87.6%) > Actin/Hsp70 (1,047,625/1,201,755 = 87.1%) > serine protease (611,784/922,464 = 66.3%) > PKABC (555,411/1,164,233 = 47.4%) > ArsC/Prdx2 (239,877/779,614 = 30.8%) (Fig. 3a), implying that AChE/BChE, as well as Actin/Hsp70, are structurally similar. In contrast, ArsC/Prdx2 are less similar. In general, amino acid grouping slightly increases the percentages of distinct Common keys and the overall ordering nearly remains the same (Fig. 3b). If we look at the distinct common keys, amino acid grouping dramatically increases distinct keys for the dataset at the root level and increases distinct keys for PKA/B/C family. In contrast, the numbers of distinct common keys decrease for Actin/Hsp70 and AChE/BChE with amino acid grouping. Amino acid grouping has no significant effect for ArsC/Prdx2 and serine proteases (Fig. 4a). If key frequency is considered, amino acid grouping increases the total number of distinct common keys for the dataset at the root level, PKA/B/C, ArsC/Prdx2, and serine proteases. The effect of amino acid grouping on Actin/Hsp70 and AChE/BChE is minimal (Fig. 4b). Amino acid grouping increases the numbers of distinct common keys, especially when key frequency is counted. This increase suggests that amino acid grouping increases structure similarity. The data also show that amino acid grouping has a minimal effect on protein clustering as judged by their functional classification (Fig. 5a) but slightly increases structural similarity (from 42.8% increased to 50.0%) as expected (Fig. 5b).

**Fig. 6:**
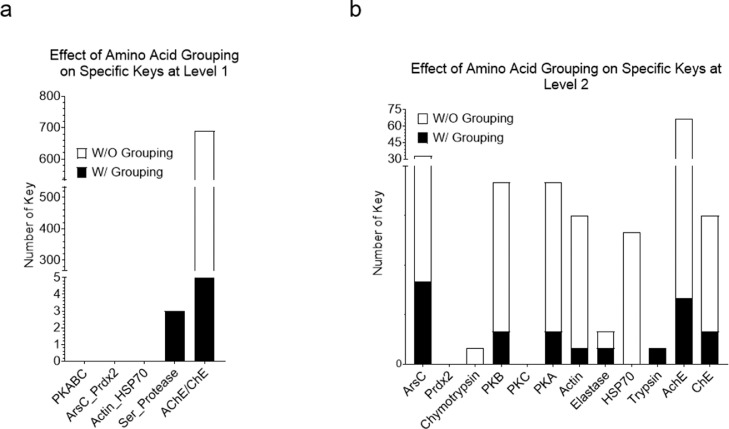
Effect of amino acid grouping on the specific keys of the structures organized in the hierarchical organization. a, At the level 1; b, At the level 2.

**Fig. 7:**
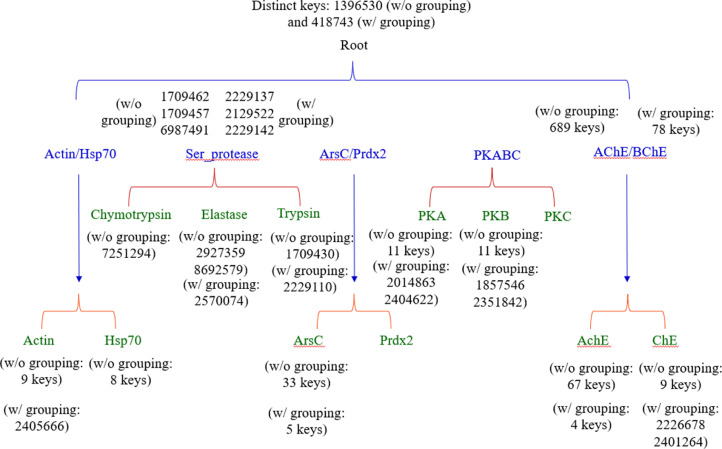
A structure-based hierarchical organization of the dataset. Numbers of the specific keys for each (sub)class or type for with and without amino acid grouping are indicated.

**Fig. 8:**
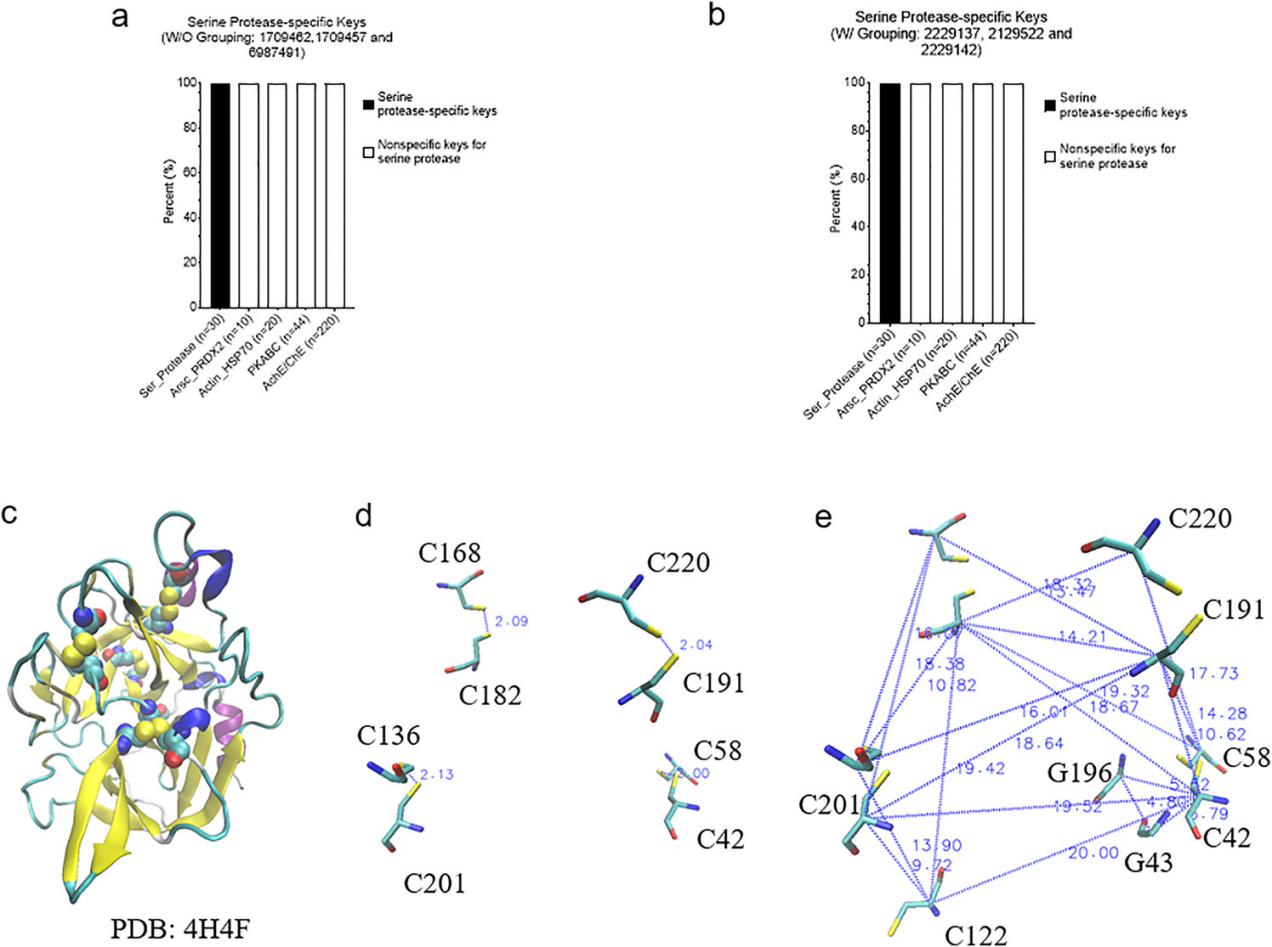
A small set of specific keys were identified and are present for serine proteases. a-b, Three specific keys for identified for without and with amino acid grouping respectively; c, Eight cysteine residues of a representative protein structure (PDB ID: 4H4F) were identified and are presented for the three keys in both with and without amino acid grouping. Four disulfide bonds were identified from these eight cysteine residues and are shown; d, The distances and cysteine positions of four disulfide bonds are shown; e, Eight triangles associated with the keys of 2129522 (Cys-Gly-Gly, 1 triangle), 2229137 (Cys-Cys-Cys, 4 triangles) and 2229142 (Cys-Cys-Cys, 3 triangles) are shown.

**Fig. 9:**
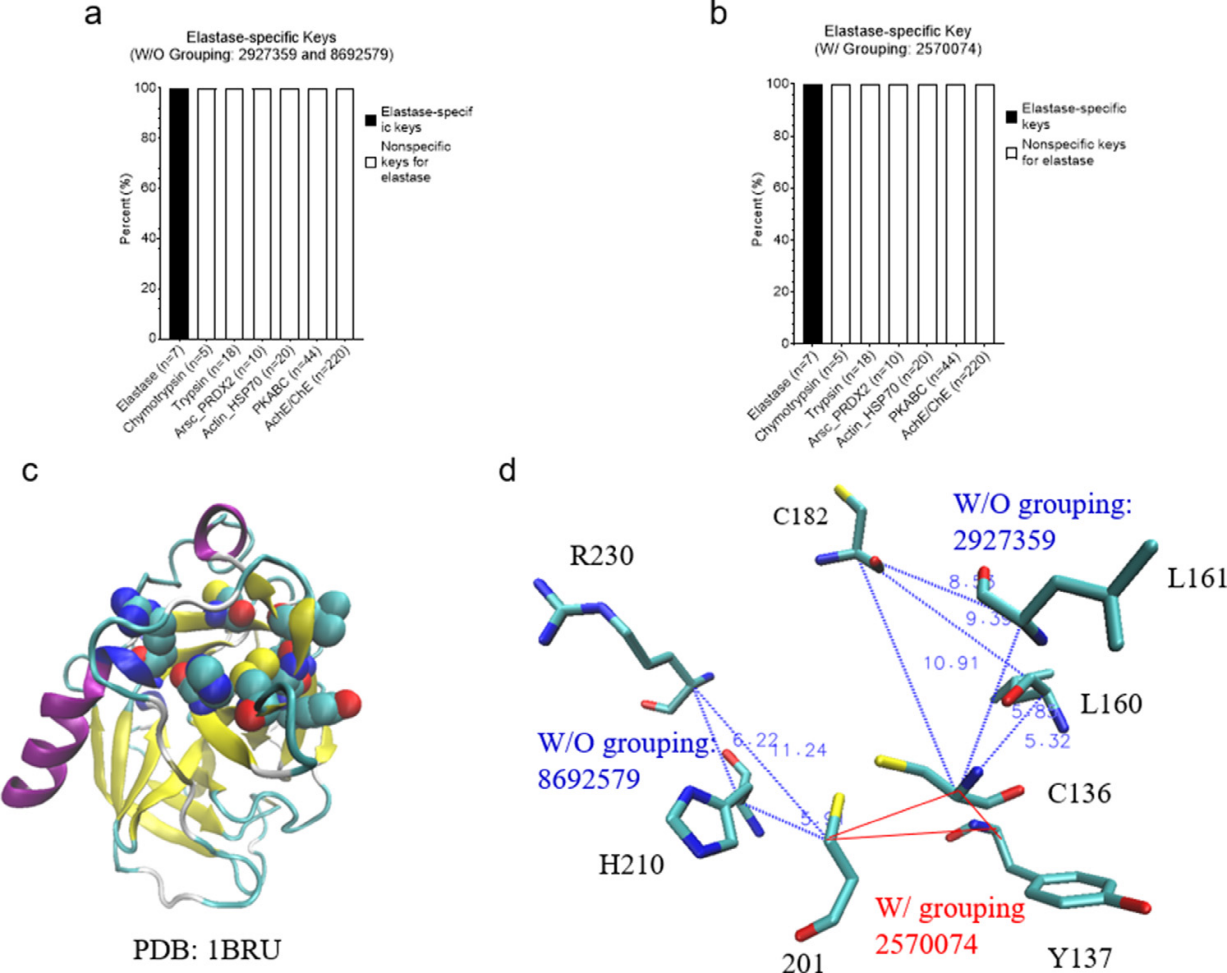
A small set of specific keys were identified and are present for elastases. a-b, Two and one specific keys for identified for without and with amino acid grouping respectively; c, The amino acids associated with the keys of 2927359, 8692579 and 2570074 are shown in VDW (PDB ID: 1BRU); d, The triangles associated with the keys of 2927359 (Cys136-L160-C182 and Cys-L161-C182), 8692579 (Cys210-H210-R230) and 2570074 (Cys136-Y137-Cys201) (PDB ID: 1BRU). With and without amino acid grouping are indicated.

We have described the effect of amino acid grouping on Common(common) keys. Next, we will focus on the discussion of the effect of amino acid grouping on specific keys that exclusively belong to a certain protein class/group. We were able to discover such keys for serine proteases and AChE/BChE but failed to find any specific keys for the classes of PKA/B/C, ArsC/Prdx2, and Actin/Hsp70 at level 1 (Fig. 6a). No specific keys were found for PKA/B/C, ArsC/Prdx2 and Actin/Hsp70 which reveals that PKA, PKB and PKC are structurally diverse. This structure diversity is also observed for ArsC and Prdx2, and Actin and Hsp70. For level 2, specific keys can be identified for all groups except Prdx2 and PKC (Fig. 6b). As expected, our data show that amino acid grouping decreases the numbers of specific keys at both level 1 and level 2. The specific keys are summarized in Fig. 7. To gain a better understanding of the specific keys and to demonstrate potential applications of our TSR-based structure comparison method in structural motif discovery with the objective of showing the difference between with and without amino acid grouping, we performed a detailed analysis on serine proteases as a case study. Three specific keys: 1709457, 1709462, and 6897491, were identified exclusively for serine proteases without amino acid grouping (Fig. 8a), and three (2129522, 2229137, and 2229142) were identified with amino acid grouping (Fig. 8b). Eight Cys residues were found in these three specific keys, with and without amino acid grouping, and these eight Cys form four disulfide bonds. Disulfide bonds require specific bond length, typically less than 2.20 Å, and it is the reason why these three keys are present exclusively for serine proteases. A representative of eight disulfide bonds from human chymotrypsin (PDB ID: 4H4F) are shown in Fig. 8C and D. The total occurrences of three specific keys of chymotrypsin (PDB ID: 4H4F) for without and with amino acid grouping are eleven and eight, respectively (Fig. 8E). Given that we successfully identified and showed the keys present exclusively in serine proteases, we next ask can we also identify the keys exclusively for chymotrypsin, trypsin, and elastase? One key (7251294 without amino acid grouping), one key/one key (1709430 without amino acid grouping/2229110 with amino acid grouping) and two keys/one key (2927359 and 8692579 without amino acid grouping/2570074 with amino acid grouping) are identified exclusively for chymotrypsin, trypsin, and elastase, respectively (Fig. 7). Uniqueness of the specific keys for elastases is demonstrated in Fig. 9a (without amino acid grouping) and 9b (with amino acid grouping). The location and geometries of the representative keys for elastase (PDB ID: 1BRU) are shown in Fig. 9c and d. Collectively, our method allows for the effective and accurate identification of similar local structures even when two structures are different at a global level. For any of the common and specific keys identified in this study that are not discussed in sufficient detail, the information including the structure datasets, and the source codes, will be made available upon request.

### 1.2 Applying amino acid grouping improves the TSR-based protein clustering

**Fig. 10:**
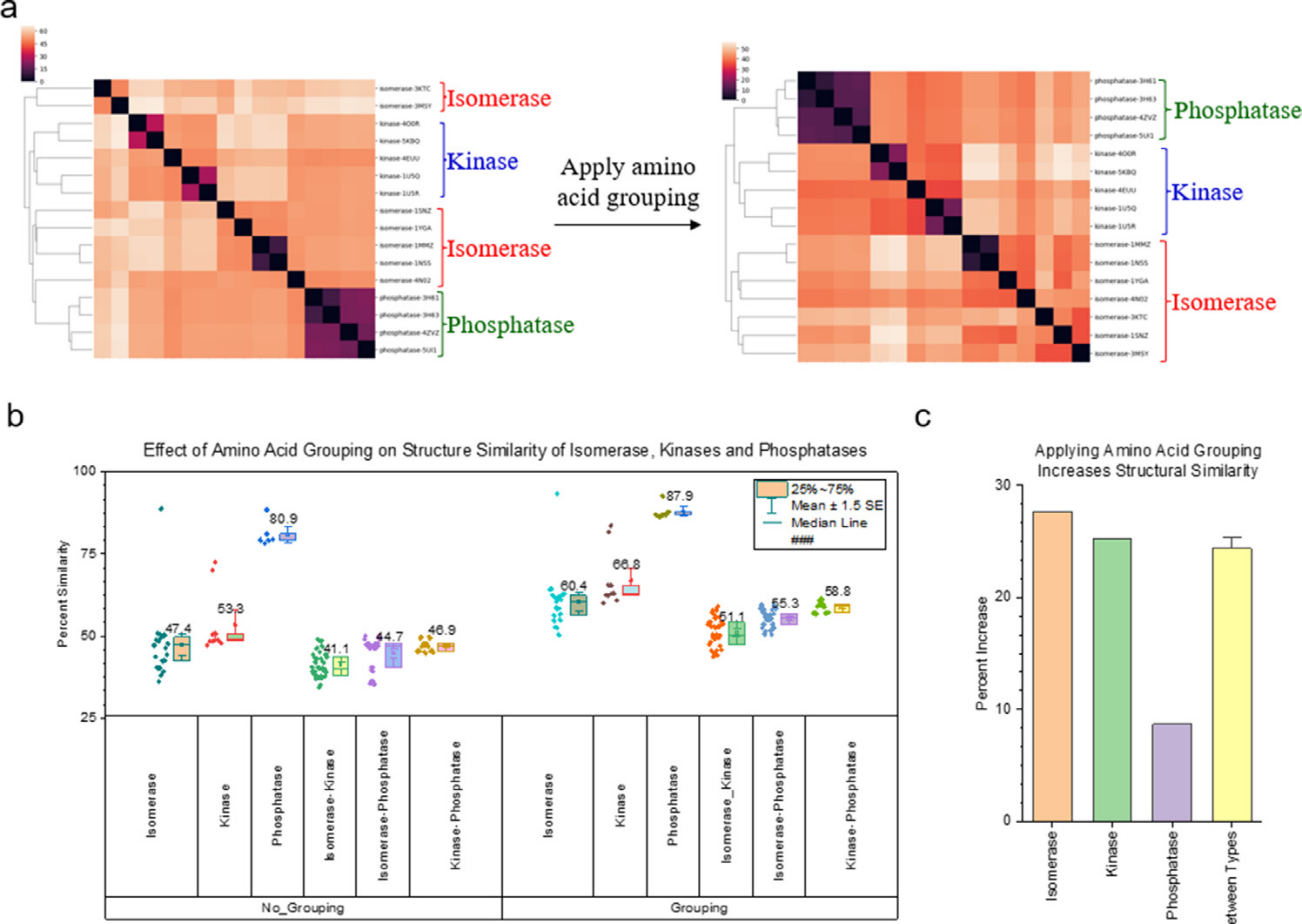
The result from a dataset containing kinases, phosphatases, and isomerases shows a clustering improvement after applying the amino acid grouping. a, The clustering maps show the protein clusters before and after amino acid grouping. The dissimilarity values are indicated in the upper left corner of the clustering maps; b, Pairwise structure similarities with and without amino acid grouping were calculated and are shown. The means are labeled and the 25 and 75 percentiles are indicated; c, Percent increases in structure similarity were calculated and are present.

**Fig. 11:**
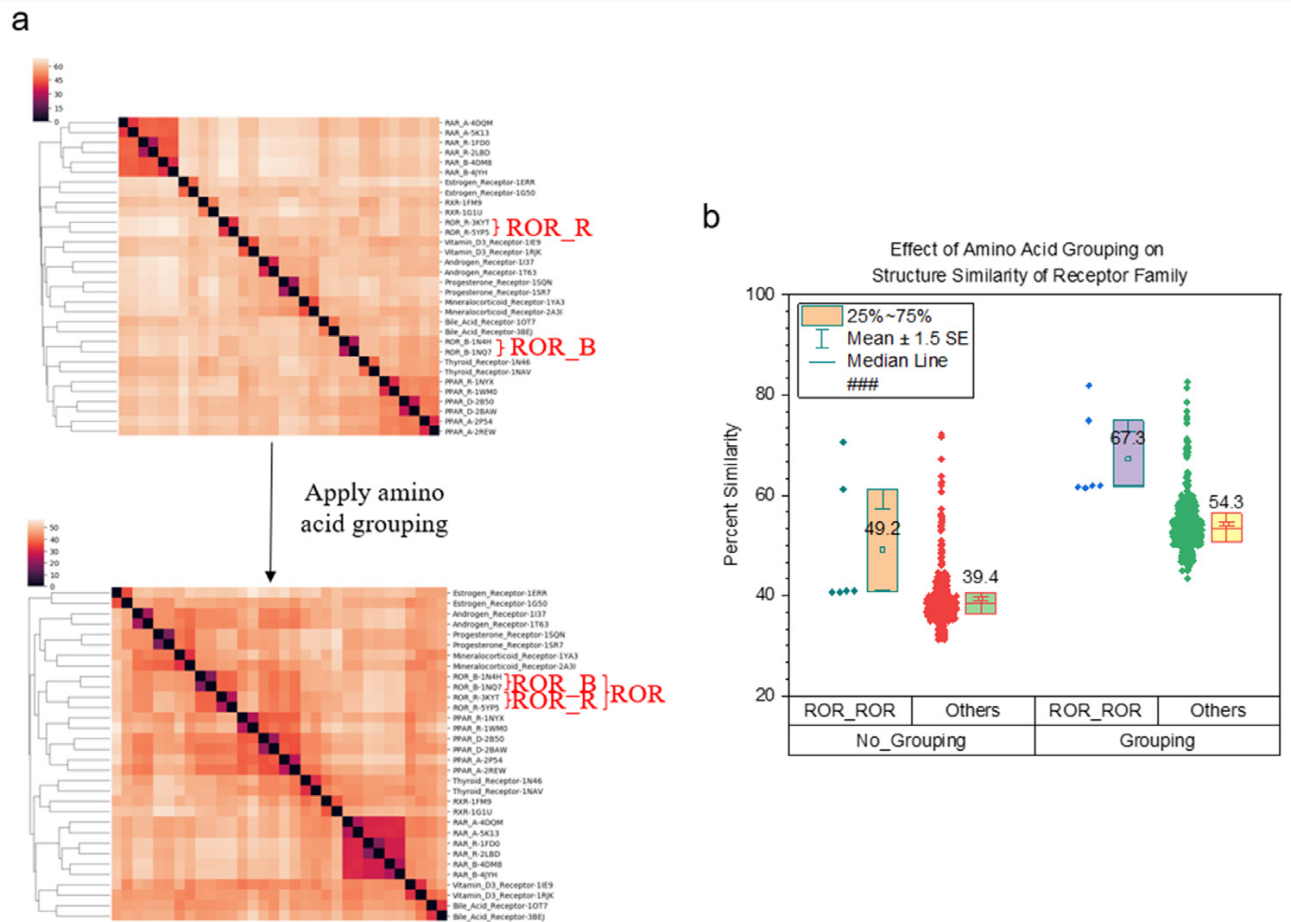
The result from a dataset containing diverse receptors shows a clustering improvement after applying the amino acid grouping. a, The clustering maps show the protein clusters before and after amino acid grouping. The dissimilarity values are indicated in the upper left corner of the clustering maps; b, Pairwise structure similarities with and without amino acid grouping were calculated and are shown. The means are labeled and the 25 and 75 percentiles are indicated.

We reported that applying amino acid grouping improves the clustering effectiveness of protein kinases A, B, and C [3]. To further demonstrate the effect of amino acid grouping on the clustering of diverse protein families, we have built two new datasets. One dataset contains protein kinases, phosphatases, and isomerase and the other dataset includes different types of protein receptors. The result from the first dataset shows that either kinases or phosphatases group together without amino acid grouping (Fig. 10a). However, there are two clusters for isomerases and one isomerase cluster is between kinases and phosphatases (Fig. 10a). The sequence alignment using Neighbor-joining algorithm shows two clusters of isomerases. One isomerase cluster is merged with kinases and the other joins with phosphatases (Supplementary Figure 1). After applying the amino acid grouping, two separated isomerase clusters get merged and become one large cluster (Fig. 10a). As expected, the amino acid grouping increases structural similarity of proteins from the same protein family as well as proteins from any two different families (Fig. 10b). The ranking of the structural similarity, which is isomerase (27.6) > kinase (25.3%) > phosphatase (8.68%) (Fig. 10c), explains why two isomerase clusters become one cluster (Fig. 10a). The structural similarity increases for protein from two different families (isomerase vs kinase, isomerase vs phosphatase, and kinase vs phosphatase) are close (Fig. 10b and c). Protein receptor families include even more diverse proteins based on sequence and structure comparisons [1]. The result from the second dataset clearly demonstrates that two well-separated ROR β and γ clusters get merged into one cluster after employing amino acid grouping (Fig. 11a). The protein sequence alignment shows ROR β and γ are grouped together (Supplementary Figure 2) that is consistent with the structural comparison after amino acid grouping. The structural similarity increases 36.8% on average for ROR β and γ subfamilies, which is comparable to all other pairwise structural comparisons (Fig. 11b).

### 1.3 Applying amino acid grouping enhances the capacity of the TSR-based method for identifying metal ion binding sites

To demonstrate the effect of amino acid grouping on motif identifications, we have focused on metal ion binding structural motifs, specifically Zn^2+^ binding sites, in this study. Zinc ions (Zn^2+^), which are the second most abundantly found within cells, are essential for life due to its function as a cofactor, signaling molecule, and structural element [4,5]. Common Zn^2+^ ligands found within proteins include cysteine (S), histidine (N), aspartate (O), and glutamate (O) residues [4]. Metallocarboxpeptidases participate in a wide range of physiological processes through cleaving C-terminal residues from peptide substrates. There are two types of metallopeptidases: *cowrins* and *funnelins*. Cowrins comprise protozoan, prokaryotic, and mammalian enzymes related to both neurolysin and angiotensin-converting enzyme with a long, deep, and narrow active-site cleft. Funnelins comprise structural relatives of the archetypal bovine carboxypeptidase A1, and feature mammalian, insect, and bacterial proteins with a shallow active-site cleft lying at the bottom of a funnel-like cavity [6]. Both cowrins and funnelins follow a common general acid and base mechanism [6]. Thermolysin [EC 3.4.24.27], originally identified in the culture broth of Bacillus thermoproteolyticus, is a thermostable zinc metalloproteinase [7]. Thermolysin has the consensus zinc-binding motif sequence, HExxH [8]. Two histidine residues of HExxH and one glutamate residue outside HExxH chelate the active-site Zn^2+^ [8]. The glutamate of HExxH is important to catalytic activity [8].

Angiotensin-converting enzyme (ACE) is ubiquitously expressed while its mammalian homologue, ACE2, is primarily expressed in the lung, heart, kidney, and testis [9]. Both ACE and ACE2 are key regulators of the renin-angiotensin system through their zinc-metallopeptidase activity on vasoactive peptides [10]. ACE2 serves as a receptor for the severe acute respiratory syndrome coronavirus 2 (SARS-CoV-2), which is responsible for the coronavirus disease 2019 (COVID-19) pandemic [10]. ACE and ACE2 contain single HEXXH zinc-binding domain [11]. Assays of ACE2 activity require the presence of zinc with high activity when its concentration is less than 10 µM [12].

**Fig. 12:**
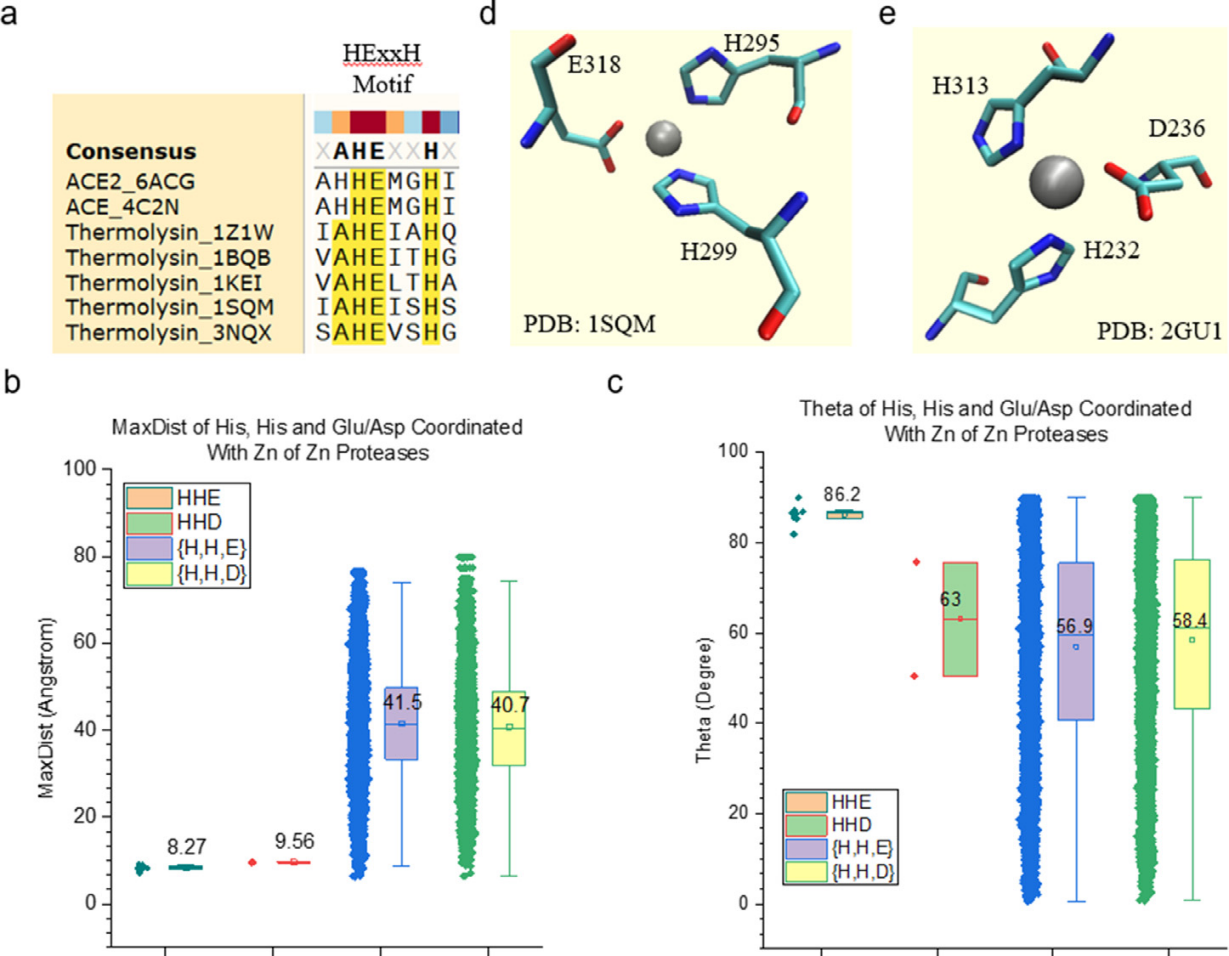
The Zn^2+^ binding sites of proteases have their unique geometries. a, The sequence alignment study shows HExxH motif of ACE, ACE2 and thermolysin; b, MaxDist values of the triangles constituted from two His and one Glu or one Asp in the Zn^2+^ binding sites as well as those not in the binding sites were calculated and are shown; c, Theta values of the triangles constituted from two His and one Glu or one Asp in the Zn^2+^ binding sites as well as those not in the binding sites were calculated and are shown; b-c, The means are labeled and the 25 and 75 percentiles are indicated; d-e, Two representative Zn^2+^ binding sites: two His and one Glu (d) and two His and one Asp (e), are shown. The PDB IDs are indicated. The amino acids are labeled and Zn^2+^ ions are shown.

**Fig. 13:**
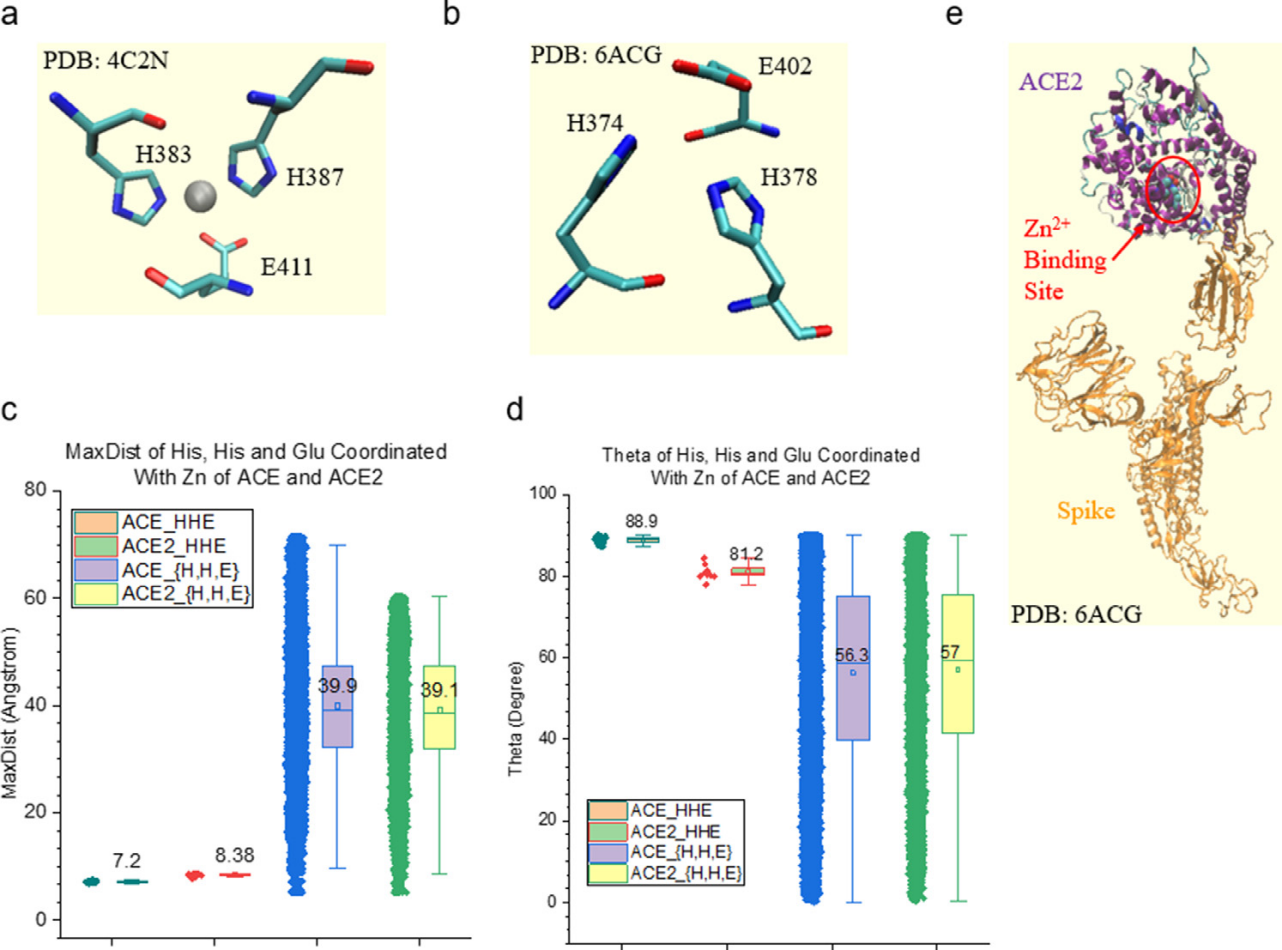
The Zn^2+^ binding sites of ACE and ACE2 have their unique geometries. a-b, Two representative Zn^2+^ binding sites of ACE (a) and ACE2 (b) are shown. The PDB IDs are indicated. The amino acids are labeled. c-d, MaxDist and Theta values of the triangles constituted from two His and one Glu in the Zn^2+^ binding sites as well as those not in the binding sites were calculated and are shown. The means are labeled and the 25 and 75 percentiles are indicated; e, A representative structure shows interactions between spike of SARS-CoV-2 and human ACE2. The PDB ID is indicated and Zn^2+^ binding site is labeled.

A Zn^2+^ binding protein dataset was prepared including diverse proteases, ACE and ACE2. Thermolysin, ACE, and ACE2 have the HExxH sequence motif (Fig. 12a), while other endopeptidases and carboxypeptidases do not have a clear HExxH motif. The Zn^2+^ binding sites comprise two histidine residues and one glutamate or one aspartate residue. For thermolysin, the two histidine residues are from the HExxH motif. In contrast, for endopeptidases and carboxypeptidases, they are not from a clear sequence motif like HExxH. It is important to notice that the glutamate in the Zn^2+^ binding site of thermolysin is not the glutamate in the HExxH motif. The Zn^2+^ binding sites of the proteases studied have similar MaxDist (Fig. 12b) and Theta (Fig. 12c) values. However, they are different from the triangles constituted from two histidine residues and either one glutamate or one aspartate residue, which do not form a Zn^2+^ binding motif (Fig. 12b and c). The representative Zn^2+^ binding motifs containing two His and one Glu or one Asp are shown in Fig. 12d and e. The Zn^2+^ binding sites of ACE and ACE2 have a similar geometry as those of proteases (Fig. 13a–c) although ACE2 has a slightly smaller Theta, on average, than ACE, possibly due to the apo-enzyme forms (No Zn^2+^ ions are in those active sites) of some ACE2. The Zn^2+^ binding site is not close to the interface between Spike and ACE2 (Fig. 13e). These results demonstrate that the TSR-based method provides a unique way for not only searching and identifying metal binding sites, but also for employing amino acid grouping to enhance the capacity of the method for systematically studying structural motifs of metal ion binding sites.

## 2 Experimental Design, Materials and Methods

### 2.1 Key generation

For every protein, C_α_ atoms from its PDB file were selected. All three edge lengths and angles of all possible triangles formed by C_α_ were calculated. For the case *without amino acid grouping*, each C_α_ of the 20 amino acids was assigned a unique integer identifier in the range (4, 5, …, 23) [2]. For the case *with amino acid grouping*, we grouped Ser and Thr together with the same integer because Ser and Thr have similar structures and functions. Similarly, we grouped and assigned the same integers for Ala and Val; Leu and Ile; Phe and Trp; Asp and Glu; Asn and Gln; and Lys and Arg. For the case with grouping, out of 20 distinct amino acids, 14 were combined to form 7 amino acid categories and the other 6 remained in a category by themselves [3]. Thus, we ended up with 13 total integer IDs, one for each amino acid category [3]. We transform the integer IDs to $l_{i1}$, $l_{i2}$ and $l_{i3}$ for vertices of triangle *i* based on the rule-based label-determination [13]. Once $l_{i1}$, $l_{i2}$ and $l_{i3}$ are determined for triangle *i*, we calculate *θ*_1_ using Equation No. 1 and *θ*_Δ_ based on *θ*_1_ values.

(1) $$\theta_{1}=\cos^{-1}\left(\left(d_{13}^{2}\right)-{\left(\frac{d_{12}}{2}\right)}^{2}-\left(d_{3}^{2}\right)/\left(2\right)\;\times \;\left(\frac{d_{12}}{2}\right)\times \left(d_{3}\right)\right)$$

$$\theta_{\Delta }=\left\{ \begin{array}{c} \theta_{1}\;\mathrm{if}\;\theta \leq 90^{\circ }\\ 180^{\circ }-\theta_{1}\;\mathrm{othe}\mathrm{rwise} \end{array}\right.$$

Where

$d_{13}$: distance between $l_{i1}$ and $l_{i3}$ for triangle *i*
$d_{12}$: distance between $l_{i1}$ and $l_{i2}$ for triangle *i*
$d_{3}$: distance between midpoint of $l_{i1}$ and $l_{i2}$, and $l_{i3}$ for triangle *i*

We refer to the value of *θ*_Δ_ as Theta and *D* as MaxDist [2]. Once labels: $l_{i1}$, $l_{i2}$, $l_{i3}$, *D* and *θ*_Δ_ are determined, we use Equation No.2 to calculate the key for each triangle.

(2) $$k=\theta_{T}d_{T}\left(l_{i1}-1\right)m^{2}+\theta_{T}d_{T}\left(l_{i2}-1\right)m+\theta_{T}d_{T}\left(l_{i3}-1\right)+\theta_{T}\left(d-1\right)+\left(\theta -1\right)$$

where

*m*: the total number of distinct labels
θ:the bin value for the class in which $\theta_{\Delta }$, the angle representative, falls; to achieve discretization, we use the Adaptive Unsupervised Iterative Discretization algorithm
$\theta_{T}$: the total number of distinct discretization levels (or bin number) for angle representative
*d*: the bin value for the class in which *D*, the length representative, falls; to achieve discretization we use the Adaptive Unsupervised Iterative Discretization algorithm
$d_{T}$*:* the total number of distinct discretization levels (or bin number) for length representative

In summary, the key value assigned to a triangle is a function of $l_{i1}$, $l_{i2}$, $l_{i3}$, *θ*_Δ_, and *D*. We will refer to the value of *θ*_Δ,_ the angle representative, as Theta and *D,* the length representative, as MaxDist. This ensures that the keys generated for the purpose of protein 3D structure comparison, while remaining rotation and translation invariant, are sensitive to scale changes. The foundation to calculate meaningful keys is based in designing an experiment that determines the numbers of bins for Theta and MaxDist. An equal width binning method will result in different numbers of bins of the triangles falling in each bin depending on whether the specified interval of values is for Theta or MaxDist. To maximize the possibility of the same or similar number of triangles in each bin and ensure that all occurrences of the same value are placed in the same bin, we used the Adaptive Unsupervised Iterative Discretization algorithm to calculate the bin boundaries [14,15]. We used numbers of bins 35 and 29 for MaxDist and Theta respectively. The details of how to determine bin boundary values and numbers of bins were reported [2]. The TSR-based structure comparison method can be integrated with molecular dynamics simulations [2,16] and experimental data [16] to achieve deeper understanding protein structure and function relations.

### 2.2 Protein structure similarity and distance calculation

We apply the Generalized Jaccard coefficient measure [17], Equation No. 3, for the calculation of similarity between two proteins.

(3) $$\text{Ja}c_{gen}=\sum_{i=1}^{n}\epsilon_{i}/\sum_{i=1}^{n}z_{i}$$

where *n* is the total number of unique keys in proteins *p_1_* and *p_2_*

Equivalence *ϵ* for a given key *k_i_* in two different proteins *p_1_* and *p_2_* is defined as $\epsilon_{i}=k_{i}^{p_{1}}\cap k_{i}^{p_{2}}$ where *∩* is defined by the minimum count of the corresponding keys*.*

Difference *z* for a given key *k_i_* in a pair of proteins is defined as $z_{i}\;=\;k_{i}^{p_{1}}\cup k_{i}^{p_{2}}$ where ∪ is defined by the maximum count of the corresponding keys. The count of a key is the number of times that key occurs (occurrence frequency) within a protein.

Once a similarity matrix is generated, the distance matrix is derived simply by taking each value in the similarity matrix and subtracting it from 1. Protein structure clustering is visualized based on Average Linkage Clustering [18]. ClustalW module built in Vector NTI [19] and SnapGene were applied to conduct pairwise sequence alignments. Structural images were prepared using the Visual Molecular Dynamics (VMD) package [20]. Sequence alignment and phylogenetic analysis were done using MEGA7 [21].

### 2.3 Preparation of protein structure datasets

In this study, we prepared one dataset of approximately 300-400 structures including ArsC/Prdx2, PKA/PKB/PKC, and AChE/BChE for investigating the effect of amino acid grouping on hierarchical clustering. The PDB IDs, chain information, and functional classification of the dataset will be available upon request. Two datasets were prepared to show improvement of protein clustering by applying amino acids grouping. The detailed information can be found in Supplementary File 1 (Isomerases, Kinases, and Phosphatases) and Supplementary File 2 (Protein Receptors). The Zn^2+^ binding protein structures have been prepared to show the effect of amino acid grouping on motif identification (Supplementary Files 3).

### 2.4 Source code

The source code is available for nonprofit use of academic research in github (https://github.com/TitliSarkar/Amino-Acid-Grouping/).

## Ethics Statement

No human subjects are involved.

No animal experiments are involved.

No data involved were collected from social media platforms.

## CRediT authorship contribution statement

**Titli Sarkar:** Conceptualization, Methodology, Software, Data curation, Writing – original draft, Visualization, Investigation, Software, Validation. **Camille R. Reaux:** Conceptualization, Methodology, Software, Data curation, Writing – original draft. **Jianxiong Li:** Conceptualization, Methodology, Software, Validation. **Vijay V. Raghavan:** Data curation, Writing – original draft, Supervision, Writing – review & editing. **Wu Xu:** Conceptualization, Methodology, Software, Data curation, Writing – original draft, Visualization, Investigation, Supervision, Writing – review & editing.

## Declaration of Competing Interest

The authors declare that they have no known competing financial interests or personal relationships which have or could be perceived to have influenced the work reported in this article.

## Data Availability

TSR Data (Original data) (TSR Data).

## Appendix Supplementary materials

Image, application 1
