# Supplementary material for: The specific applications of the TSR-based method in identifying Zn2+ binding sites of proteases and ACE/ACE2
Source: Data Brief. 2022 Sep 23;45:108629. doi: 10.1016/j.dib.2022.108629 (PMC9679521; doi:10.1016/j.dib.2022.108629)
Supplement: Supplementary file 1 [file mmc1.pdf]

Figure Legend

Supplementary Figure 1. Phylogenetic study of kinases, phosphatases and isomerases. The phylogenetic analysis was conducted using Molecular Evolutionary Genetics Analysis (MEGA) software. A multiple sequence analysis was first conducted using ClutalW. The aligned proteins were then used to generate a Neighbor-Joining phylogenetic tree.

Supplementary Figure 2. Phylogenetic study of diverse receptors. The phylogenetic analysis was conducted using Molecular Evolutionary Genetics Analysis (MEGA) software. A multiple sequence analysis was first conducted using ClutalW. The aligned proteins were then used to generate a Neighbor-Joining phylogenetic tree.

Supplementary Figure 1

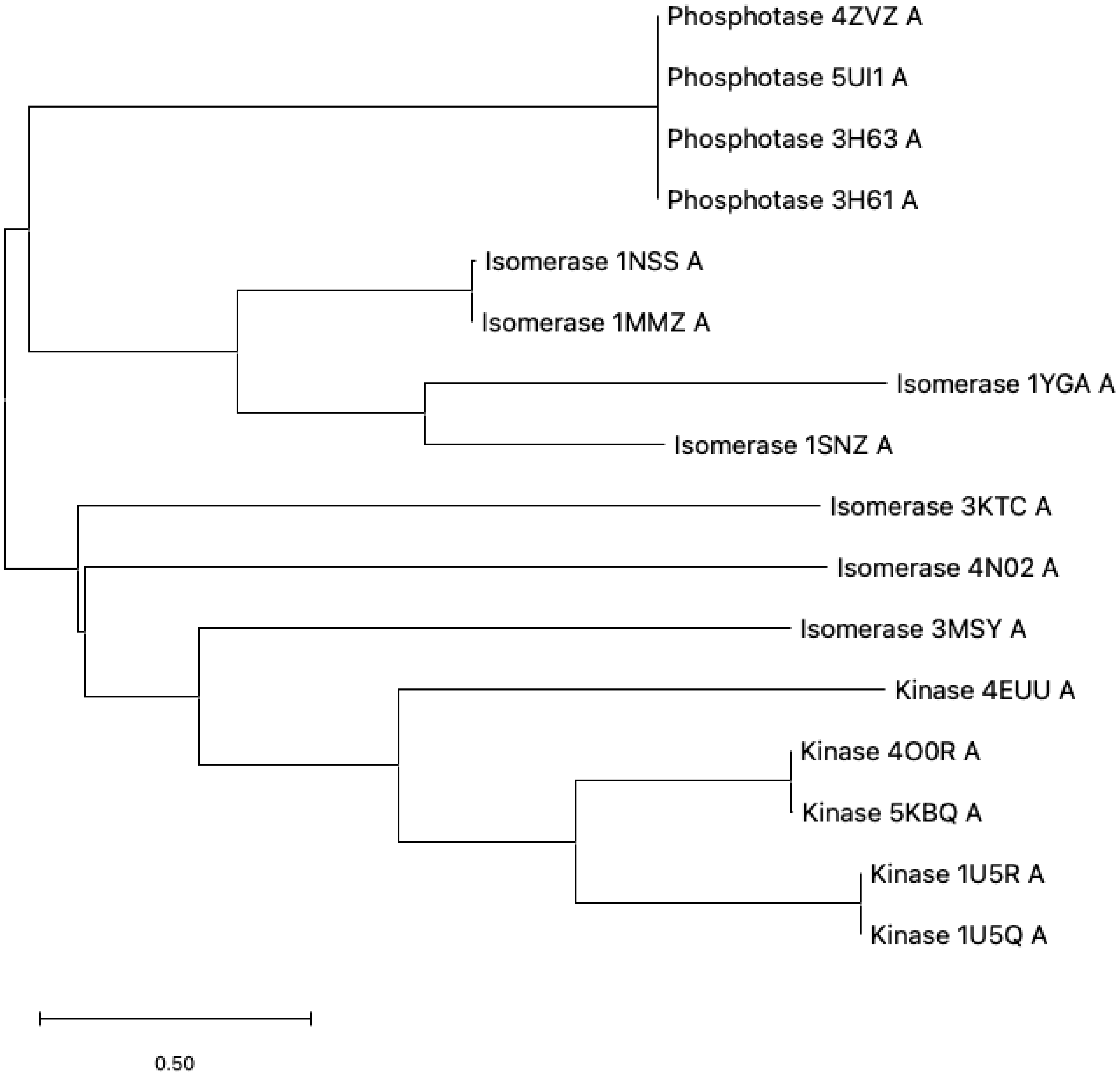

Supplementary Figure 2

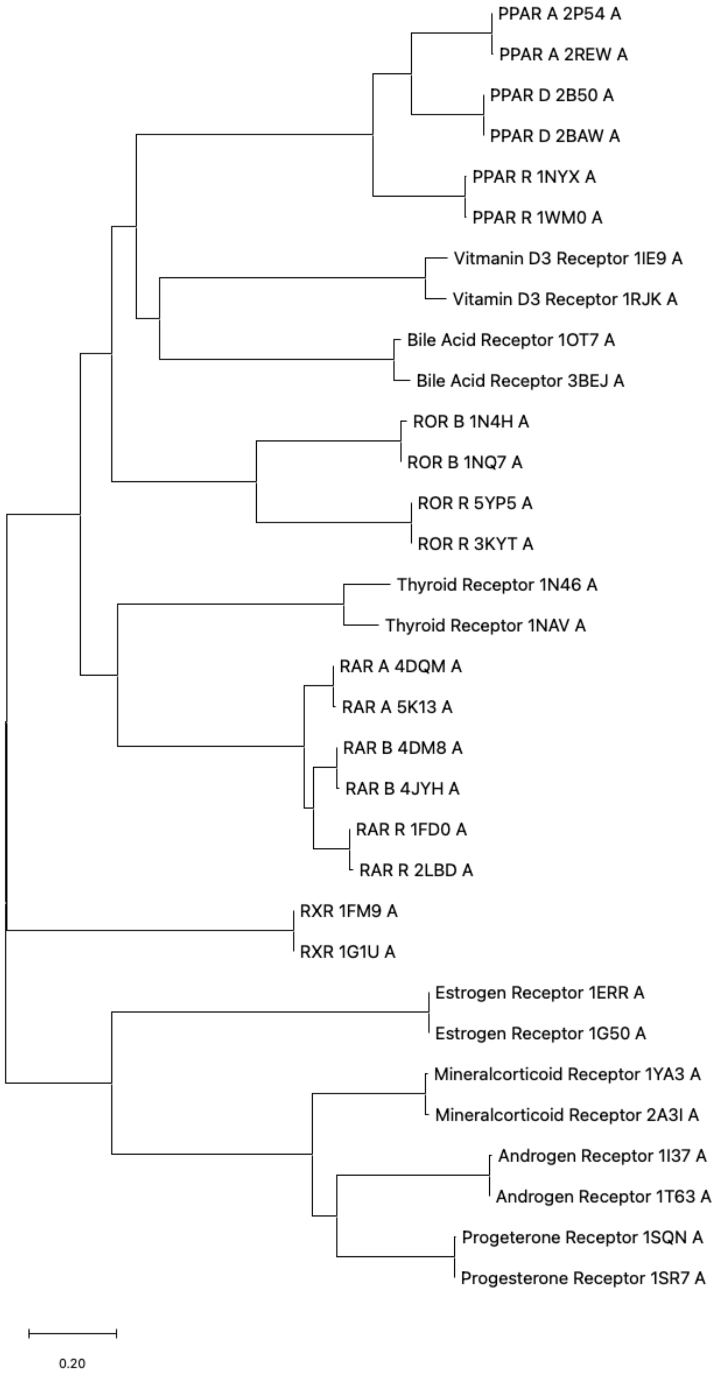

Supplementary File 1

protein	chain	group
3H61	A	phosphatase
3H63	A	phosphatase
4ZVZ	A	phosphatase
5UI1	A	phosphatase
4O0R	A	kinase
5KBQ	A	kinase
4EUU	A	kinase
1U5R	A	kinase
1U5Q	A	kinase
1NSS	A	isomerase
1YGA	A	isomerase
4N02	A	isomerase
3KTC	A	isomerase
3MSY	A	isomerase
1SNZ	A	isomerase
1MMZ	A	isomerase

Supplementary File 2

protein	chain	group
2P54	A	PPAR_A
2REW	A	PPAR_A
2B50	A	PPAR_D
2BAW	A	PPAR_D
1NYX	A	PPAR_R
1WM0	X	PPAR_R
4DQM	A	RAR_A
5K13	A	RAR_A
4DM8	A	RAR_B
4JYH	A	RAR_B
1FD0	A	RAR_R
2LBD	A	RAR_R
1N4H	A	ROR_B
1NQ7	A	ROR_B
5YP5	A	ROR_R
3KYT	A	ROR_R
1FM9	A	RXR
1G1U	A	RXR
1N46	A	Thyroid_Receptor
1NAV	A	Thyroid_Receptor
1IE9	A	Vitamin_D3_Receptor
1RJK	A	Vitamin_D3_Receptor
1I37	A	Androgen_Receptor
1T63	A	Androgen_Receptor
1OT7	A	Bile_Acid_Receptor
3BEJ	A	Bile_Acid_Receptor
1ERR	A	Estrogen_Receptor
1G50	A	Estrogen_Receptor
1YA3	A	Mineralocorticoid_Receptor
2A3I	A	Mineralocorticoid_Receptor
1SQN	A	Progesterone_Receptor
1SR7	A	Progesterone_Receptor

Supplementary File 3

protein	chain	group
3NQX	A	Thermolysin
1DMT	A	Thermolysin
1S4B	P	Thermolysin
1SQM	A	Thermolysin
1KEI	A	Thermolysin
1BQB	A	Thermolysin
1GE6	A	Endopeptidase
1YME	A	Carboxypeptidase
1Z1W	A	Thermolysin
2GU1	A	Carboxypeptidase
1O86	A	ACE_Human
1O8A	A	ACE_Human
1R42	A	ACE2_Human
1R4L	A	ACE2_Human
1UZE	A	ACE_Human
1UZF	A	ACE_Human
2AJF	A	ACE2_Human
2C6F	A	ACE_Human
2C6N	A	ACE_Human
2IUL	A	ACE_Human
2IUX	A	ACE_Human
2OC2	A	ACE_Human
2XY9	A	ACE_Human
2XYD	A	ACE_Human
2YDM	A	ACE_Human
3BKK	A	ACE_Human
3BKL	A	ACE_Human
3L3N	A	ACE_Human
3NXQ	A	ACE_Human
3SCI	A	ACE2_Human
3SCJ	A	ACE2_Human
4APH	A	ACE_Human
4APJ	A	ACE_Human
4BXK	A	ACE_Human
4BZR	A	ACE_Human
4BZS	A	ACE_Human
4C2N	A	ACE_Human
4C2O	A	ACE_Human
4C2P	A	ACE_Human
4C2Q	A	ACE_Human
4C2R	A	ACE_Human
4CA5	A	ACE_Human
4CA6	A	ACE_Human
4UFA	A	ACE_Human
4UFB	A	ACE_Human

5AM8	A	ACE_Human
5AM9	A	ACE_Human
5AMA	A	ACE_Human
5AMB	A	ACE_Human
5AMC	A	ACE_Human
6EN5	A	ACE_Human
6EN6	A	ACE_Human
6F9R	A	ACE_Human
6F9T	A	ACE_Human
6F9U	A	ACE_Human
6F9V	A	ACE_Human
6H5W	A	ACE_Human
6H5X	A	ACE_Human
6LZG	A	ACE2_Human
6M0J	A	ACE2_Human
6QS1	A	ACE_Human
6TT1	A	ACE_Human
6TT3	A	ACE_Human
6TT4	A	ACE_Human
6VW1	A	ACE2_Human
3KBH	A	ACE2_Human
6ACG	D	ACE2_Human
6ACJ	D	ACE2_Human
6ACK	D	ACE2_Human
6CS2	D	ACE2_Human
